# γ-Aminobutyric Acid Enhances Heat Tolerance Associated with the Change of Proteomic Profiling in Creeping Bentgrass

**DOI:** 10.3390/molecules25184270

**Published:** 2020-09-18

**Authors:** Zhou Li, Weihang Zeng, Bizhen Cheng, Ting Huang, Yan Peng, Xinquan Zhang

**Affiliations:** Department of Grassland Science, College of Animal Science and Technology, Sichuan Agricultural University, Chengdu 611130, China; zengwh0123@163.com (W.Z.); Chengbizhengrass@163.com (B.C.); huangting88win@163.com (T.H.); pengyanlee@163.com (Y.P.)

**Keywords:** antioxidant, GABA, heat shock protein, osmotic adjustment, signal transduction, transcription factor

## Abstract

γ-Aminobutyric acid (GABA) participates in the regulation of adaptability to abiotic stress in plants. The objectives of this study were to investigate the effects of GABA priming on improving thermotolerance in creeping bentgrass (*Agrostis stolonifera*) based on analyses of physiology and proteome using iTRAQ technology. GABA-treated plants maintained significantly higher endogenous GABA content, photochemical efficiency, performance index on absorption basis, membrane stability, and osmotic adjustment (OA) than untreated plants during a prolonged period of heat stress (18 days), which indicated beneficial effects of GABA on alleviating heat damage. Protein profiles showed that plants were able to regulate some common metabolic processes including porphyrin and chlorophyll metabolism, glutathione metabolism, pyruvate metabolism, carbon fixation, and amino acid metabolism for heat acclimation. It is noteworthy that the GABA application particularly regulated arachidonic acid metabolism and phenylpropanoid biosynthesis related to better thermotolerance. In response to heat stress, the GABA priming significantly increased the abundances of Cu/ZnSOD and APX4 that were consistent with superoxide dismutase (SOD) and ascorbate peroxidase (APX) activities. The GABA-upregulated proteins in relation to antioxidant defense (Cu/ZnSOD and APX4) for the reactive oxygen species scavenging, heat shock response (HSP90, HSP70, and HSP16.9) for preventing denatured proteins aggregation, stabilizing abnormal proteins, promoting protein maturation and assembly, sugars, and amino acids metabolism (PFK5, ATP-dependent 6-phosphofructokinase 5; FK2, fructokinase 2; BFRUCT, β-fructofuranosidase; RFS2, galactinol-sucrose galactosyltransferase 2; ASN2, asparagine synthetase 2) for OA and energy metabolism, and transcription factor (C2H2 ZNF, C2H2 zinc-finger protein) for the activation of stress-defensive genes could play vital roles in establishing thermotolerance. Current findings provide an illuminating insight into the new function of GABA on enhancing adaptability to heat stress in plants.

## 1. Introduction

With global warming, heat-induced losses in an agricultural economy will further extend worldwide [1]. Multiple mechanisms of plants adapting to high temperature environments have been investigated in the past 50 years. Undoubtedly, protein expression and reprograming are very important for plants to acclimate to heat stress. For example, heat shock proteins (HSPs) accumulation play a central role in the acquisition of thermotolerance in different plant species, since the HSPs family can help to promote the degradation of misfolded proteins and prevent misfolded proteins from aggregating in cells under heat stress [2]. Nowadays, isobaric tags for relative and absolute quantitation (iTRAQ)-based proteome provides an illuminating insight into the change of protein profiles and key metabolic pathways in plants subjected to heat stress. The study of [3] found that the alteration of proteins associated with antioxidant defense, photosynthesis, and HSPs played positive roles in enhancing heat adaptation in grape (*Vitis vinifera*). Heat stress damaged cell membranes and photosynthesis along with significant regulation of proteins involved in abiotic stress defense, photosynthesis, and signal transduction in leaves of *Potentilla Fruticosa* [4]. Differential abundance of proteins participating in stress defense, energy, and nucleotide metabolism, and signal transduction was related to the adaptation to heat stress in mustard (*Brassica juncea*) plants [5]. These findings demonstrated that the analysis of protein profiles was an important strategy for better understanding of the thermotolerance in plants differing in heat tolerance.

Amino acids accumulation and metabolism are also crucial adaptive responses to abiotic stress in plants [6]. Recent studies highlight the role of γ-aminobutyric acid (GABA) in affecting tolerance to abiotic stress in plants [7]. It has been reported about beneficial effects of GABA on regulating nitrogen balance, antioxidant, osmotic adjustment (OA), and amino acids accumulation when plants undergone abiotic stress [8]. *Arabidopsis* calmodulin mutants became sensitive to heat stress because of the disturbance in the accumulation of GABA and other metabolites in GABA shunt under heat stress [9]. Exogenous GABA treatment could effectively alleviate the growth inhibition of heat-sensitive mung bean (*Vigna radiata*) plants when they were subjected to heat stress [10]. Antioxidant capacity and water balance regulated by exogenous GABA helped rice (*Oryza sativa*) seedlings to acclimate heat stress [11]. Enhanced GABA accumulation and metabolism contributed to the alleviation of heat-induced leaf senescence associated with significant increase in chlorophyll synthesis in white clover (*Trifolium repens*) [12]. Previous studies also found that foliar application of GABA significantly improved the thermotolerance of creeping bentgrass (*Agrostis stolonifera*) in relation to organic metabolites accumulation and upregulation of genes encoding antioxidant enzymes, stress-defensive proteins, and transcription factors [13,14]. However, GABA-regulated the change of protein profiles associated with thermotolerance is not well documented in plants.

Grasses can be divided into two main types according to the optimal growth temperature and climatic conditions. One is cold-season grass, such as creeping bentgrass that adapts to cold and humid climate with the optimal growth temperature between 15 °C and 24 °C. Another is warm-season grass, such as Bermudagrass (*Cynodon dactylon*) that is able to growth prolifically under the temperature range of 25 °C to 35 °C. Heat stress is one of the most critical abiotic stresses limiting the cultivation and utilization of cool-season grass worldwide [15]. The creeping bentgrass is widely used for establishing high quality of turf such as putting green in golf course and the sport turf in lawn tennis due to its fine texture and tolerance to low mowing than other turfgrass [16]. Although some GABA-induced physiological responses to heat stress have been demonstrated in previous studies, stress-defensive proteins accumulation and comprehensive protein profiling regulated by the GABA have not been investigated in plants under heat stress. We hypothesized that the exogenous application of GABA could significantly increase endogenous GABA that acted as a positive regulator of physiology, proteins accumulation, and relevant metabolic pathways associated with heat tolerance in plants. Thus, the objectives of this study were to analyze the effects of root priming with GABA on change of protein profiles and relevant metabolic pathways associated with thermotolerance in leaves of creeping bentgrass. Physiological and proteomic analyses will reveal the important function of GABA on regulating heat tolerance in cool-season grass.

## 2. Results

### 2.1. Physiological Changes Affected by Heat Stress and GABA Application

**Figure 1:**
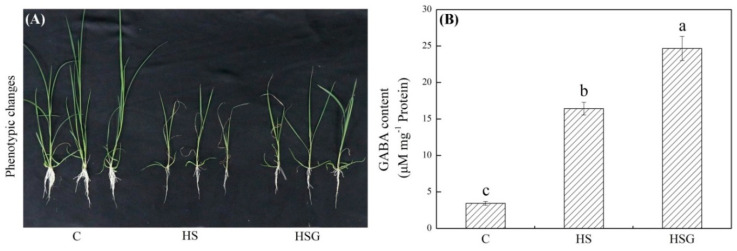
Effects of exogenous γ-aminobutyric acid (GABA) on (**A**) phenotypic changes and (**B**) endogenous GABA content in leaf of creeping bentgrass under heat stress. Vertical bars indicate ± SE of mean (*n* = 4) and different letters above columns indicate significant differences (*p* ≤ 0.05). C, control (normal condition); HS, heat stress; HSG, heat-stressed plants pretreated with GABA.

**Figure 2:**
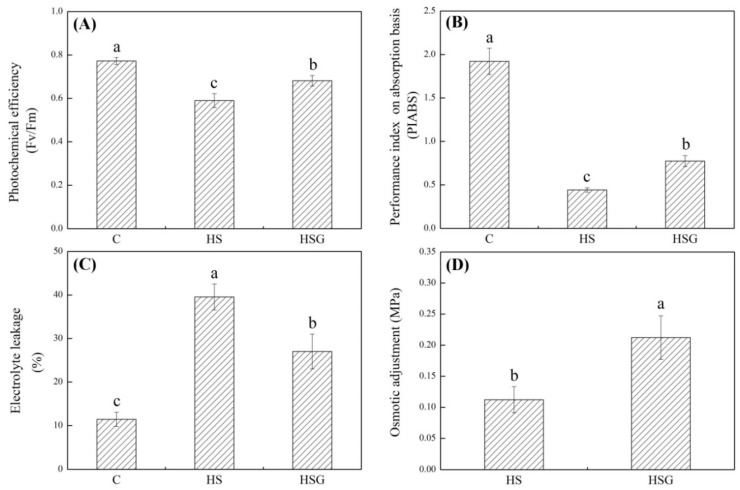
Effects of exogenous γ-aminobutyric acid (GABA) on (**A**) photochemical efficiency (Fv/Fm), (**B**) performance index on absorption basis (PIABS), (**C**) osmotic adjustment (OA), and (**D**) electrolyte leakage (EL) in leaf of creeping bentgrass under heat stress. Vertical bars indicate ± SE of mean (*n* = 4) and different letters above columns indicate significant differences (*p* ≤ 0.05). C, control (normal condition); HS, heat stress; HSG, heat-stressed plants pretreated with GABA.

A prolonged period of heat stress significantly inhibited plant growth, but the inhibitory effect was weaker in the GABA-treated plants as compared to that in the untreated plants (Figure 1A). Endogenous GABA content was kept at a lower level under non-stress conditions, and heat stress significantly increased GABA accumulation in leaves (Figure 1B). Under heat stress, plants with GABA priming exhibited 50% increase in GABA content than untreated plants (Figure 1B). Photochemical efficiency (Fv/Fm) and performance index on absorption basis (PIABS) decreased significantly in both of GABA-pretreated and untreated plants in response to heat stress. The heat-stressed plants pretreated with GABA (HSG) treatment showed 16% or 75% increase in Fv/Fm or PIABS than the heat stress (HS) treatment, respectively (Figure 2A,B). The electrolyte leakage (EL) increased by 246% in the HS or by 136% in the HSG as compared to that in the control (C), respectively (Figure 2C). GABA-pretreated plants also exhibited significantly higher OA than untreated plants under heat stress (Figure 2D).

**Figure 3:**
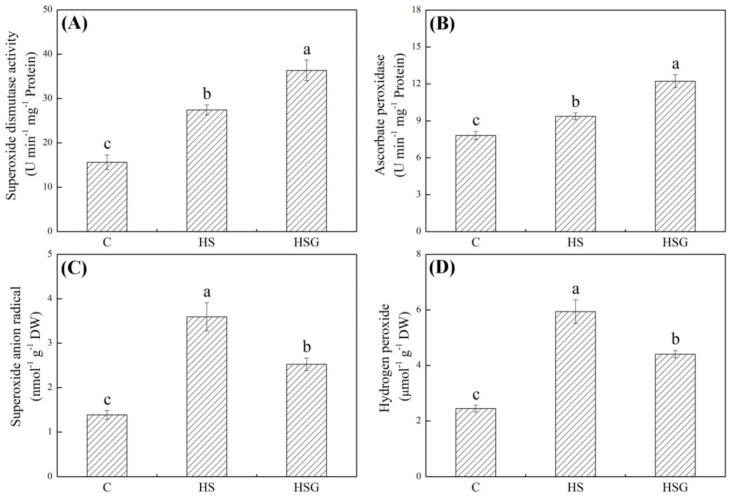
Effects of exogenous γ-aminobutyric acid (GABA) on (**A**) superoxide dismutase (SOD) activity, (**B**) ascorbate peroxidase (APX) activity, (**C**) superoxide anion radical (O_2_^−^), and (**D**) hydrogen peroxide (H_2_O_2_) in leaf of creeping bentgrass under heat stress. Vertical bars indicate ± SE of mean (*n* = 4) and different letters above columns indicate significant differences (*p* ≤ 0.05). C, control (normal condition); HS, heat stress; HSG, heat-stressed plants pretreated with GABA.

Heat stress significantly enhanced superoxide dismutase (SOD) and ascorbate peroxidase (APX) activities in plants with or without GABA application (Figure 3A,B). GABA-pretreated plants exhibited 32% or 30% increase in SOD or APX activity than untreated plants under heat stress, respectively (Figure 3A,B). O_2_^−^ and H_2_O_2_ significantly accumulated when all plants suffered from heat stress (Figure 3C,D). The GABA priming significantly alleviated heat-induced increases in O_2_^−^ and H_2_O_2_ content in leaves, since the HSG showed 30% or 26% decrease in O_2_^−^ or H_2_O_2_ content than the HS, respectively (Figure 3C,D).

### 2.2. Changes of Proteins Profiling Affected by GABA and Heat Stress

**Figure 4:**
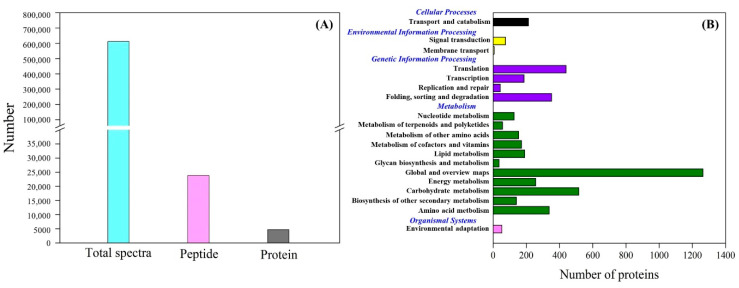
(**A**) Number of total spectra, total peptides, and total proteins, and (**B**) clusters of orthologous groups (COGs) analysis of total proteins in leaf of creeping bentgrass.

**Figure 5:**
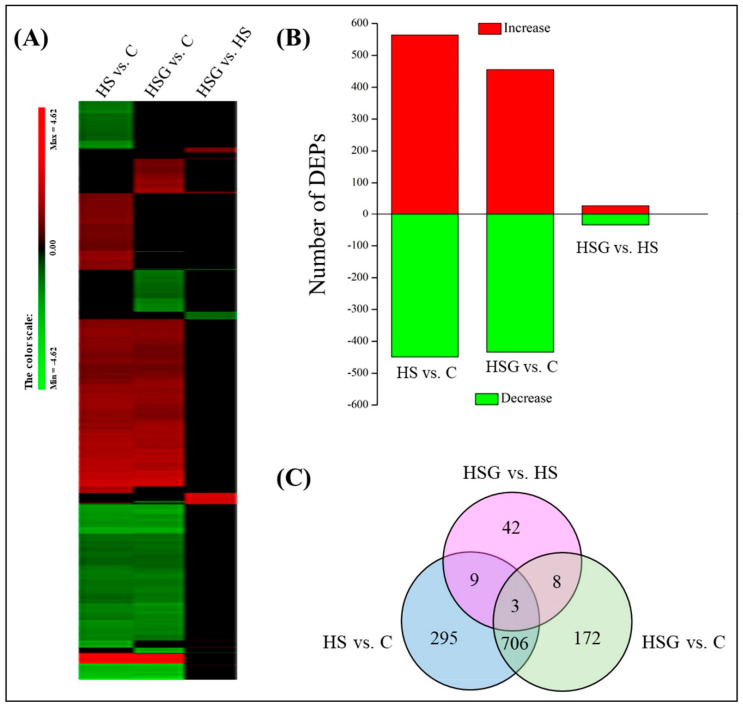
(**A**) A clustering analysis of differentially expressed proteins (DEPs), (**B**) number of DEPs, and (**C**) Venn diagram of DEPs in leaves of creeping bentgrass in response to exogenous γ-aminobutyric acid (GABA) and heat stress. C, control (normal condition); HS, heat stress; HSG, heat-stressed plants pretreated with GABA.

**Figure 6:**
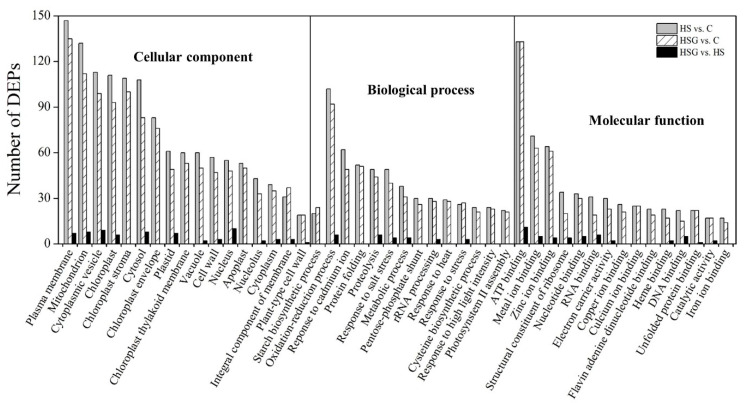
Gene Ontology (GO) analysis of differentially expressed proteins (DEPs) in leaf of creeping bentgrass in response to exogenous γ-aminobutyric acid (GABA) and heat stress. C, control (normal condition); HS, heat stress; HSG, heat-stressed plants pretreated with GABA.

Figure 4A showed total spectra, total peptide, and total proteins that were identified in leaves based on the proteome. More than 5000 proteins were identified in leaves (Figure 4A). For cellular processes, these proteins are mainly involved in transport and catabolism. For the environmental information processing, most of proteins are involved in signal transduction and membrane transport. For the genetic information processing, these identified proteins were associated with translation, transcription, and folding, sorting and degradation. For the metabolism, the majority of proteins were involved in carbohydrate, amino acid, energy, and lipid metabolism (Figure 4B). The application of GABA altered the expression pattern of differentially expressed proteins (DEPs) in leaves, as reflected by differences in DEPs between HS vs. C and HSG vs. C (Figure 5A). A total of 1013 DEPs, including 564 upregulated and 449 downregulated DEPs, were observed in HS vs. C. There were 455 upregulated and 434 downregulated DEPs in HSG vs. C (Figure 5B). Only 3 common DEPs were detected in HSG vs. HS, HS vs. C, and HSG vs. C (Figure 5C). HS vs. C and HSG vs. C had 706 common DEPs. A total of 295, 172, or 42 DEPs was detected specifically in HS vs. C, HSG vs. C, or HSG vs. HS, respectively (Figure 5C). Figure 6 showed the Gene Ontology (GO) analysis of identified DEPs in HS vs. C, HSG vs. C, and HSG vs. HS. For the cellular component, most of DEPs located in plasm membrane, mitochondrion, cytoplasmic vesicle, chloroplast, chloroplast stroma, and cytosol. For the biological process, a lot of DEPs were involved in oxidation-reduction process, response to cadmium ion, protein folding, and proteolysis. For the molecular function, the majority of DEPs had the function of ATP binding, metal ion binding, zinc ion binding, nucleotide binding, and RNA binding (Figure 6).

**Figure 7:**
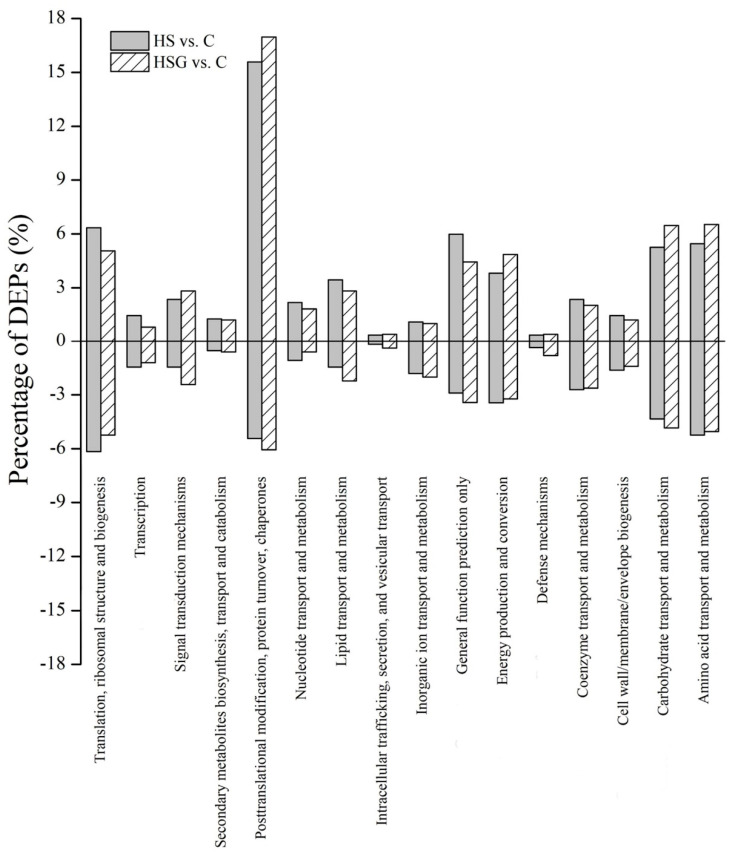
Kyoto Encyclopedia of Genes and Genomes (KEGG) analysis of identified differentially expressed proteins (DEPs) in leaf of creeping bentgrass in response to exogenous γ-aminobutyric acid (GABA) and heat stress. C, control (normal condition); HS, heat stress; HSG, heat-stressed plants pretreated with GABA.

**Figure 8:**
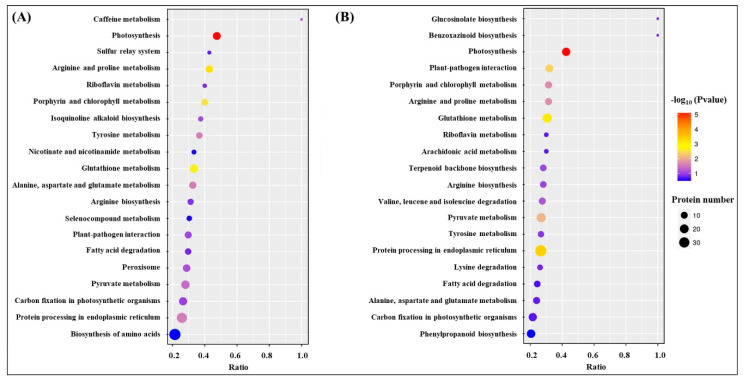
The effect of heat stress without (HS vs. C) and with (HSG vs. C) exogenous γ-aminobutyric acid (GABA) application on differentially expressed proteins (DEPs) within each functional category based on the clusters of orthologous groups (COGs) in leaf of creeping bentgrass. (**A**) HS vs. C and (**B**) HSG vs. C. C, control (normal condition); HS, heat stress; HSG, heat-stressed plants pretreated with GABA.

Kyoto Encyclopedia of Genes and Genomes (KEGG) analysis showed that the most of DEPs were involved in translation-ribosomal structure and biogenesis, posttranslational modification-protein turnover-chaperones, energy production and conversion, and carbohydrate or amino acid transport and metabolism (Figure 7). HSG vs. C had more DEPs involved in posttranslational modification-protein turnover-chaperones and carbohydrate or amino acid transport and metabolism then HS vs. C (Figure 7). Based on functional category of clusters of orthologous groups (COGs), HS vs. C and HSG vs. C included some common metabolic pathways such as photosynthesis, porphyrin and chlorophyll metabolism, riboflavin metabolism, glutathione metabolism, fatty acid degradation, pyruvate metabolism, carbon fixation in photosynthetic organisms, amino acids metabolism, and protein processing in endoplasmic reticulum (Figure 8). The arachidonic acid metabolism and phenylpropanoid biosynthesis were only observed in HSG vs. C among the top 20 pathways (Figure 8B).

### 2.3. The Interaction Network of DEPs

**Figure 9:**
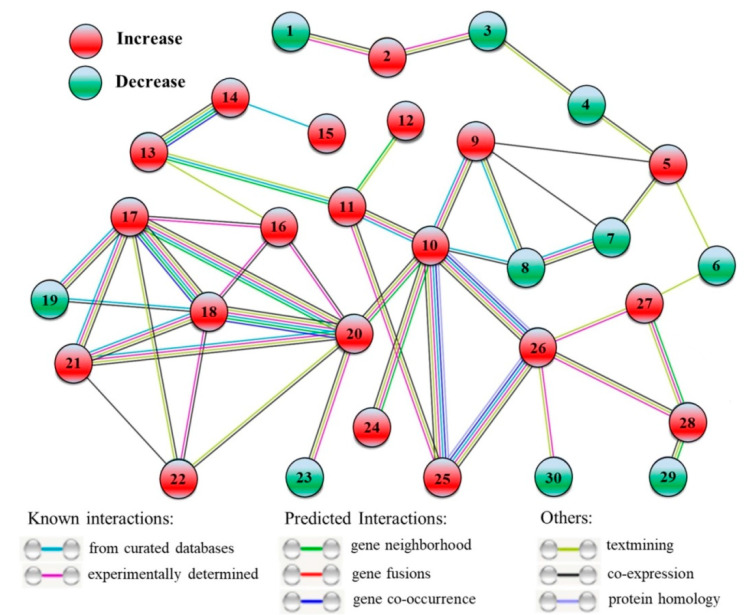
Interaction network of the identified differentially expressed proteins (DEPs) when HSG compared to HS (HSG vs. HS). This network shows only known and direct interactions among identified DEPs. C, control (normal condition); HS, heat stress; HSG, heat-stressed plants pretreated with γ-aminobutyric acid (GABA). 1, RPA2; 2, SWI2; 3, CHB3; 4, EMB140; 5, C2H2 ZNF; 6, AGO1; 7, SFU2af-A; 8, SFU2af-B; 9, BCLP; 10, HSP70b; 11, PFK5; 12, ASN2; 13, FK2; 14, BFRUCT; 15, RFS2; 16, RGG; 17, 40SRP-S3; 18, 40SRP-S14; 19, ETIF5; 20, 60SRP-L4; 21, 60SRP-L28; 22, BTF3; 23, Tim44; 24, HSP16.9; 25, HSP90; 26, HSP70-2; 27, Cu/ZnSOD; 28, APX4; 29, ETFBETA; 30, DNL-type ZFP.

A total of 30 DEPs were included in the interaction network of proteins from HSG vs. HS (Figure 9). The 20 out of 30 DEPs were upregulated (2, SWI2, Switch 2; 5, C2H2 ZNF, C2H2 zinc-finger protein; 9, BCLP, β-Catenin-like protein; 10, HSP70b, Heat shock protein 70b; 11, PFK5, ATP-dependent 6-phosphofructokinase 5; 12, ASN2, Asparagine synthetase 2; 13, FK2, Fructokinase 2; 14, BFRUCT, β-Fructofuranosidase; 15, RFS2, Galactinol-sucrose galactosyltransferase 2; 16, RGG, RGG repeats nuclear RNA binding protein A; 17, 40SRP-S3, 40S ribosomal protein S3; 18, 40SRP-S14, 40S ribosomal protein S14; 20, 60SRP-L4, 60S ribosomal protein L4; 21, 60SRP-L28, 60S ribosomal protein L28; 22, BTF3, Basic transcription factor 3; 24, HSP16.9, Heat shock protein 16.9; 25, HSP90, Heat shock protein 90; 26, HSP70-2, Heat shock protein 70-2; 27, Cu/ZnSOD, Cu/Zn Superoxide dismutase; 28, APX4, Ascorbate peroxidase 4) and the left 10 DEPs were down-regulated (1, RPA2, Replication protein A; 3, CHB3, SWI/SNF complex subunit SWI3D; 4, EMB140, Embryo defective 140; 6, AGO1, Protein argonaute 1; 7, SFU2af-A, Splicing factor U2af large subunit A; 8, SFU2af-B, Splicing factor U2af small subunit B; 19, ETIF5, Eukaryotic translation initiation factor 5; 23, Tim44, Tim44-related protein; 29, ETFBETA, Electron transfer flavoprotein subunit beta; 30, DNL-type ZFP, DNL-type zinc finger protein) (Figure 9). The basic information of these DEPs, including short name, full name, and fold change of these DEPs, is recorded in Table S1.

## 3. Discussion

Typical symptoms of heat damage include accelerated senescence and significant decline in photosynthesis [17]. It has been proved that chlorophyll a fluorescence parameters could act as important indicators of abiotic stress [18]. The study of [19] found that heat stress caused damage to photosystem II leading to significant decline in Fv/Fm, but plants could switch photosystem through driving cyclic electron flow for adapting to heat environment. Based on the comparative analysis of two hybrids of colonial (*Agrostis capillaris*) × creeping bentgrass differing in heat tolerance, heat stress-induced leaf senescence was mainly related to accelerated chlorophyll degradation [20]. Similarly, chlorophyll b-deficient wheat (*Triticum aestivum*) mutants showed lower photoprotective responses as compared to wild types under high temperature environment [21]. An previous study in creeping bentgrass has shown that the GABA exhibited positive function in alleviating heat-induced chlorophyll loss and photoinhibition [13]. The recent study also found that enhanced GABA accumulation and metabolism helped to maintain chlorophyll synthesis and decrease chlorophyll degradation in white clover, thereby mitigating the decline in photosynthesis during heat stress [12]. In current study, the creeping bentgrass with GABA priming maintained better photochemical efficiency (Fv/Fm), photosynthetic performance (PIABS), cell membrane stability, and OA than untreated plants in response to a prolonged period of heat stress, indicating that the beneficial function of GABA on alleviating heat damage could be associated with better photoprotective response and water regulation.

Damage effects of heat stress are closely associated with proteins degradation, denaturation, and metabolic disturbance in plants [22,23]. Plants are apt to activate or regulate more metabolic processes to acclimate the constantly increasing heat damage [24]. The analysis of protein profiles demonstrated that more DEPs were detected in HS vs. C as compared to HSG vs. C, which could indicate that GABA-pretreated creeping bentgrass maintained better cellular homeostasis than the plants without GABA priming for the same duration of heat stress. The functional category of COGs showed that heat stress and heat plus GABA application induced some common metabolic processes such as photosynthesis, porphyrin and chlorophyll metabolism, glutathione metabolism, pyruvate metabolism, carbon fixation, and amino acids metabolism. Changes of these metabolic pathways associated with thermotolerance have been reported in many plant species including creeping bentgrass [3,5,25]. Interestingly, the phenylpropanoid biosynthesis and arachidonic acid metabolism were only observed in HSG vs. C among the top 20 metabolic pathways. Secondary metabolites including lignin and flavonoids generated from the phenylpropanoid biosynthesis and metabolism are beneficial to the tolerance to abiotic stress in plants [26]. Transcriptional analysis has also proved that phenylpropanoid biosynthesis was an important pathway contributing to GABA-induced thermotolerance in creeping bentgrass [27]. In addition, the arachidonic acid acting as a signaling molecule regulates stress adaptability in plants [28,29,30]. Thus, GABA-regulated thermotolerance could be related to arachidonic acid metabolism and phenylpropanoid biosynthesis in creeping bentgrass.

Enhanced antioxidant capacity is one of the most basic protective responses when plants go through harsh environments, since abiotic stress including high temperature accelerated reactive oxygen species (ROS) production and accumulation leading to oxidative damage [31]. Effects of GABA on improving antioxidant defense have been reported in many plant species including creeping bentgrass under different abiotic stresses. GABA priming induced a significant increase in SOD activity that helped to alleviate O_2_^−^ accumulation in black pepper (*Piper nigrum*) under water stress [32]. Exogenous GABA protected maize (*Zea mays*) seedlings from oxidative damage by increasing SOD and APX activities during salt stress [33]. In response to heat stress, the GABA application also could significantly increase SOD and APX activities associated with significant decline in oxidative damage in creeping bentgrass and rice [11,13]. Current results showed that lower O_2_^−^ and H_2_O_2_ accumulation and significant increases in the proteins abundance of Cu/ZnSOD and APX4 as well as enhanced SOD and APX activities were detected in HSG vs. HS. These findings support that the GABA-enhanced antioxidant defense is not only involved in the improvement of SOD and APX activities, but also associated with the abundance of SOD and APX proteins in creeping bentgrass under heat stress. It is noteworthy that our previous study found that heat stress significantly inhibited SOD activity in leaves of creeping bentgrass at 21, 28, and 35 d of heat stress [13]. However, SOD activity significantly increased at 18 d of heat stress in current study. The possible mechanism was that SOD activity was also affected by the duration and severity of stress damage in plants. In response to an earlier phase of heat stress, plants have ability to activate antioxidant defense to acclimate to oxidative damage, but when plants suffered from severe stress at a later phase, the antioxidant defense declined due to stress-induced systemic damage.

Five major HSP families have been found in plants including HSP100, HSP90, HSP70, HSP60, and small HSP (sHSP) with molecular weight between 12 to 42 kDa. The HSP70 can prevent denatured proteins aggregation and refold no-native proteins, and the sHSP functions as molecular chaperone in stabilizing abnormal proteins during abiotic stress [2]. In addition to the function in promoting proteins ripening and assembly, the HSP90 also regulates extensive stress signal transduction in plants [34]. Previous studies supported beneficial functions of HSP90, HSP70, and sHSP on thermotolerance in creeping bentgrass. For examples, the study of [35] found that creeping bentgrass cultivar ‘L-93′ with better heat tolerance could accumulate more HSP70 than heat-sensitive cultivar ‘Penncross’ after 14 days of heat stress. Cytokinin alleviated heat-induced leaf senescence in relation to the HSP70 accumulation in leaves of creeping bentgrass [25]. Foliar spray of medium level of nitrogen (7 kg·ha^−1^) further improved heat-induced HSP90, HSP70, and sHSP accumulation in leaves contributing to enhanced thermotolerance in creeping bentgrass [36]. Transcriptional analyses also proved that exogenous GABA could significantly up-regulate *HSP17.8*, *HSP26.7*, *HSP70*, and *HSP90.1-b1* expression in leaves of creeping bentgrass under high temperature condition, whereas the application of biosynthesis inhibitor of GABA depressed heat-induced increases in these genes expression [37]. The results were consistent with our current findings, which indicated that GABA-induced the accumulation of HSP90, HSP70, and HSP16.9 could play central roles in establishing heat tolerance in creeping bentgrass.

The interaction network of DEPs in HSG vs. HS also identified some other proteins that could attribute to GABA-induced thermotolerance in creeping bentgrass. ATP-dependent 6-phosphofructokinase (PFK) is a rate-limiting enzyme of glycolysis, and the fructokinase (FK) acts as an allosteric activator of PFK [38,39]. β-Fructofuranosidase (BFRUCT) is involved in hydrolysis of disaccharide into monosaccharide, and galactinol-sucrose galactosyltransferase (RFS) catalyzes the synthesis of raffinose that is an important soluble carbohydrate in plants [40,41]. Asparagine synthetase (ASN) is known to be involved in the synthesis of l-asparagine from l-aspartate and this metabolic process plays important roles in nitrogen assimilation, recycling, and signal transduction in plants [42]. The study of [10] found that exogenous supplementary GABA ameliorated thermotolerance of mung bean through enhancing the accumulation of sugars and amino acids. The analysis of metabolome has also shown that the acquirement of thermotolerance could be induced by GABA associated with amino acids and sugars accumulation for better maintenance of OA and energy supply in creeping bentgrass [13]. As a transcription factor, C2H2 zinc-finger protein (C2H2 ZNF) regulates the tolerance to abiotic stresses through acting downstream genes in plants [43]. Transgenic *Brassica juncea* overexpressing a *C2H2 ZNF* significantly improved tolerance against drought, salt, and oxidative stress [44]. However, the relationship between C2H2 ZNF and GABA-induced stress tolerance has not been reported in plants by far. In response to a prolonged period of heat stress, the GABA-induced increases in PFK5, FK2, BFRUCT, RFS2, and ASN2, in leaves could play positive effects on improving sugars and amino acids metabolism for OA and energy supply, and the application of GABA also increased C2H2 ZNF that could regulate downstream stress-defensive genes in creeping bentgrass under heat stress. However, the regulatory role of GABA in activating C2H2 ZNF to affect thermotolerance of plants needs to be further elucidated in future study.

## 4. Materials and Methods

### 4.1. Plant Material and Treatments

The test material was creeping bentgrass cv. Penncross. Seeds (4 g/m^2^) were sown uniformly in seedling trays (25 cm length, 15 cm width, and 10 cm height) that was placed in a growth chamber (21/18 °C (day/night), 65% relative humidity, and 700 µmol·m^−2^·s^−1^ PAR). Seeds germinated in distilled water for 10 d and then were watered by using Hoagland’s solution [45] for 14 days. Seedlings were transplanted carefully from the seedling tray to Hoagland’s solution for 6 days to acclimate to hydroponic cultivation. Thirty-day-old seedlings were divided into two groups: one grew in Hoagland’s solution containing 0.5 mM GABA and another grew in normal Hoagland’s solution without GABA supplement for 2 days. All plants were then transferred to new Hoagland’s solution without GABA. For treatment C, the plants without GABA priming were placed in normal growth chamber that provided normal growth condition as described above for 18 days. For treatments HS and HSG, the plants without and with GABA priming were placed in high temperature growth chamber that provided 38/33 °C (day/night), 65% relative humidity, and 700 µmol·m^−2^·s^−1^ PAR for 18 days. All Hoagland’s solutions were refreshed every day and aerated using aeration pumps (115 V, 60 Hz, Tetra^®^ Blacksburg, VA, USA) to avoid the change in concentration and hypoxia. For each treatment, three independent biologic replicates were used for the analysis of proteomics and other parameters.

### 4.2. Physiological Parameters

For osmotic potential (OP), fresh leaves (0.2 g) were fully hydrated in distilled water at 4 °C for 8 h. These leaves were removed from water and then frozen in liquid nitrogen for 20 min. Leaves were thawed and cells saps were pressed from leaves. The 10 mL of the sap was inserted into a sampling chamber of an osmometer (Wescor, Logan, UT, USA) to detect osmolality (c). The OP was converted according to MPa = −c × 2.58 × 10^−3^ [46]. OA was calculated as the difference in OP between heat-stressed leaves and normal leaves. The endogenous GABA content was detected by using the method of enzyme-linked immunosorbent assay (the Assay Kit, Shanghai enzyme-linked Biotechnology Co., Ltd., Shang Hai, China) according to manufacturer’s instructions. For leaf electrolyte leakage (EL), fresh leaves (0.2 g) were collected and immediately submerged in 40 mL of distilled water for 24 h. The initial solution conductance (C_initial_) was detected by using a conductivity meter (Model 32, Yellow Spring, OH, USA). The leaves were then autoclaved at 120 °C for 20 min and cooled down to 22 °C. The C_max_ of killed leaves was measured. The EL (%) = C_initial_ /C_max_ × 100% [47]. For the determination of photochemical efficiency (Fv/Fm) and performance index on absorption basis (PIABS), a chlorophyll fluorescence system (Pocket PEA, Hansatech, United Kingdom) was used. Leaves were nipped by leaf clips for 20 min dark adaptation and the parameters were recorded.

### 4.3. Reactive Oxygen Species and Antioxidant Enzyme Activities

For the determination of superoxide anion radical (O_2_^−^) content, fresh leaves (0.1 g) were homogenated with 1.5 mL of 65 mM cold phosphate buffer (pH 7.8) and centrifuged at 4 °C for 30 min at 10,000× *g*. The 0.5 mL cold phosphate buffer, 0.5 mL of the supernatant, and 0.1 mL of 10 mM hydrochloride were mixed together and then incubated in 25 °C water bath for 20 min. The 2 mL of 58 mM sulfonamide and 7 mM alpha-naphthylamine solution was added and placed in 25 °C water bath for 20 min. The 2 mL of chloroform was added and mixed evenly. The absorption of chloroform phase was measured at 530 nm [48]. For H_2_O_2_ content, fresh leaves (0.1 g) were homogenized with 5 mL of 0.1% TCA and centrifuged at 12,000× *g* for 20 min. The 0.5 mL of supernatant was mixed with 0.5 mL of 10 mM potassium phosphate and 1 mL of KI. The absorption of solution was measured at 390 nm [49]. For antioxidant enzyme extracts, fresh leaves (0.2 g) were ground with 3 mL of 50 mM cold phosphate buffer (pH 7.8) and then centrifuged at 12,000× *g* for 30 min at 4 °C. The supernatant is enzyme extracts. The 0.05 mL of enzyme extract was mixed with 1.5 mL of reaction solution consisting of 50 mM phosphate buffered saline (PBS), 195 mM methionine, 60 μM riboflavin, and 1.125 mM NBT for the determination of superoxide dismutase (SOD) activity [50] or containing 10 mM AsA, 5 mM H_2_O_2_, 0.003 mM EDTA, and 100 mM PBS for the determination of ascorbate peroxidase (APX) activity [51]. The change in absorbance was recorded at 290 nm after every 10 s for 1 min.

### 4.4. Protein Extraction, iTRAQ Labeling, and Identification of Proteins

For iTRAQ-based protein profiles, the protein extraction, iTRAQ labeling, and identification were clearly recorded in our previous study [52]. Identified proteins were screened as the differentially expressed proteins (DEPs) if fold change (FC) of proteins ≥1.2 or ≤0.83 and *p* < 0.05. Gene Ontology (GO) analysis were conducted by using the interproscan-5 program against the non-redundant protein database [53]. The Clusters of Orthologous Groups (COG) and Kyoto Encyclopedia of Genes and Genomes (KEGG) were used to analyze the protein family and pathways [54]. The probable interacting partners were predicted using the STRING-db server [55].

### 4.5. Statistical Analysis

The significance was analyzed by using the general linear model procedure of Statistical Product and Service Solutions 24 (SPSS Institute, 2018, Cary, NC, USA). The least significant difference (LSD) at the *p* ≤ 0.05 was used for testing significant differences.

## 5. Conclusions

Physiological changes including photochemical efficiency, membrane stability, oxidative damage, and OA indicated beneficial effects of GABA on alleviating heat damage in creeping bentgrass. Based on proteomics, plants were able to regulate some common metabolic processes such as photosynthesis, porphyrin and chlorophyll metabolism, glutathione metabolism, pyruvate metabolism, carbon fixation, and amino acids metabolism to acclimate to a prolonged period of heat stress. The GABA particularly regulated arachidonic acid metabolism and phenylpropanoid biosynthesis related to thermotolerance. In response to heat stress, the GABA improved Cu/ZnSOD, APX4, HSP90, HSP70, and HSP16.9 accumulation, which could play vital roles in scavenging ROS, preventing denatured proteins aggregation, stabilizing abnormal proteins, and promoting proteins ripening and assembly for establishing thermotolerance. In addition, the GABA-induced increases in PFK5, FK2, BFRUCT, RFS2, and ASN2 were associated with sugars and amino acids metabolism for OA and energy supply, and the application of GABA also enhanced C2H2 ZNF accumulation in relation to the regulation of downstream stress-defensive genes in creeping bentgrass under heat stress.

## Supplementary Materials

The following are available online, Table S1: Basic information of key differential expressed proteins (DEPs) in HSG vs. HS based on the analysis of interaction network.
