# Supplementary material for: γ-Aminobutyric Acid Enhances Heat Tolerance Associated with the Change of Proteomic Profiling in Creeping Bentgrass
Source: Molecules. 2020 Sep 18;25(18):4270. doi: 10.3390/molecules25184270 (PMC7571209; doi:10.3390/molecules25184270)
Supplement: Supplementary file 1 [file molecules-25-04270-s001.zip › molecules-905112-supplementary.pdf]

Table S1 Basic information of key differential expressed proteins (DEPs) in HSG vs. HS based on the analysis of interaction network.

No.	Full name	Short name	Fold change	Significance
1	Replication protein A	RPA2	0.78	down
2	Switch 2	SWI2	1.60	up
3	SWI/SNF complex subunit SWI3D	CHB3	0.82	down
4	Embryo defective 140	EMB140	0.81	down
5	C2H2 zinc-finger protein	C2H2 ZNF	1.31	up
6	Protein argonaute 1	AGO1	0.78	down
7	Splicing factor U2af large subunit A	SFU2af-A	0.75	down
8	Splicing factor U2af small subunit B	SFU2af-B	0.75	down
9	beta-Catenin-like protein	BCLP	1.22	up
10	Heat shock protein 70b	HSP70b	1.54	up
11	ATP-dependent 6-phosphofructokinase 5	PFK5	1.28	up
12	Asparagine synthetase 2	ASN2	1.21	up
13	Fructokinase 2	FK2	1.35	up
14	β-Fructofuranosidase	BFRUCT	1.23	up
15	Galactinol-sucrose galactosyltransferase 2	RFS2	1.22	up
16	RGG repeats nuclear RNA binding protein A	RGG	1.28	up
17	40S ribosomal protein S3	40SRP-S3	1.39	up
18	40S ribosomal protein S14	40SRP-S14	1.26	up
19	Eukaryotic translation initiation factor 5	ETIF5	0.78	down
20	60S ribosomal protein L4	60SRP-L4	1.33	up
21	60S ribosomal protein L28	60SRP-L28	1.22	up
22	Basic transcription factor 3	BTF3	1.27	up
23	Tim44-related protein	Tim44	0.80	down
24	Heat shock protein 16.9	HSP16.9	1.27	up
25	Heat shock protein 90	HSP90	1.51	up
26	Heat shock protein 70-2	HSP70-2	1.43	up
27	Cu/Zn Superoxide dismutase	Cu/ZnSOD	1.41	up
28	Ascorbate peroxidase 4	APX4	1.36	up
29	Electron transfer flavoprotein subunit beta	ETFBETA	0.82	down
30	DNL-type zinc finger protein	DNL-type ZFP	0.74	down
